# Characterization of Path Loss and Large-Scale Fading for Rapid Intervention Team Communication in Underground Parking Garages

**DOI:** 10.3390/s19112431

**Published:** 2019-05-28

**Authors:** Seppe Van Brandt, Robbe Van Thielen, Jo Verhaevert, Tanja Van Hecke, Hendrik Rogier

**Affiliations:** IDLab, Department of Information Technology, Ghent University-imec, Technologiepark-Zwijnaarde 126, 9052 Gent, Belgium; Robbe.VanThielen@UGent.be (R.V.T.); Jo.Verhaevert@UGent.be (J.V.); Tanja.VanHecke@UGent.be (T.V.H.); Hendrik.Rogier@UGent.be (H.R.)

**Keywords:** rapid intervention team, breadcrumb system, underground parking garage, sensor network, reliability, shadowing, path loss

## Abstract

This paper reports the characterization of the 2.45-GHz-ISM-band radio wave propagation channel. Specifically, measurements were performed in an underground parking garage, with the aim of optimizing breadcrumb systems for a Rapid Intervention Team application. The effects of the high penetration loss and large reflections by the concrete reinforced building structure on the path loss and the large-scale fading were studied. Based on the analysis of the wireless channel, critical points for reliable communication between members of a Rapid Intervention Team were identified. In particular, attention was paid to dealing with large, spatially confined signal losses due to shadowing, the anticipation of corner losses and the ability of the system to operate on multiple floors.

## 1. Introduction

A Rapid Intervention Team (RIT) is a specialized team consisting of at least two firefighters. Their primary focus is the search and rescue of both firefighters and civilians inside a fire [1,2]. Reliable communication is essential to an RIT. In some environments, however, wireless communication is particularly challenging and setting up a direct communication link between the RIT inside the burning building and the base station outside is not possible. To ensure a reliable wireless connection when entering large buildings, an ad hoc breadcrumb network is proposed and further developed in [3,4,5,6,7]. However, such developed breadcrumb systems must remain reliable in even the most challenging of environments that an RIT can face. One of these environments is an underground parking garage, since, firstly, the RIT has to move downwards while fire tends to spread upwards. Secondly, an underground parking garage consists mostly of thick (reinforced) concrete walls and pillars. These structures not only contain the fire and heat inside [2], but make radio wave propagation very difficult [8,9].

A breadcrumb system is based upon the deployment of new communication nodes by the users as they are making their way through the building. Thereby, these “breadcrumbs” establish an ad hoc communication infrastructure. The time spent by the RIT on deploying breadcrumbs should be minimal. Moreover, since the team operates in very stressful conditions, the operations that they are required to perform should be as simple as possible. For these reasons, automatic deployment mechanisms have been developed. An essential part of these automatic mechanisms is knowing when and where the next breadcrumb has to be dropped. Algorithms have been developed in which the link quality between the breadcrumb chain and the users is continuously monitored. When the link quality drops under a certain threshold, the dropping mechanism is activated and a new node is added to the breadcrumb chain automatically. It has been shown that this algorithm works rather well in normal building environments [3,4,5,6,7]. In environments similar to underground parking garages, however, the thick reinforced concrete walls will lead to high penetration losses and severe multipath due to the large reflections on the walls, floors and ceilings.

In Section 2, the measurement hardware and setup are discussed. A brief explanation of the performed data analysis is given in Section 3. The measurement results are described and discussed in Section 4 and Section 5, respectively. The paper is finalized with a conclusion in Section 6.

## 2. Measurement Setup and Hardware

Several representative scenarios were chosen to include all possible paths an RIT may encounter during a real life operation, taking into account their specific movements. Furthermore, the specific channel sounding hardware applied during the different experiments aimed to mimic the breadcrumb nodes by limiting the equipment’s size and the applied power for the transmitter (Tx) and receivers (Rx).

### 2.1. Hardware

The measurement setup consisted of two NI USRP (Universal Software Radio Peripheral) N210s [10], which are Software Defined Radios (SDRs) both utilizing a VERT2450 [11] dipole antenna and operating at 2.45 GHz with 18 dBm radiated power. A carrier frequency of 2.45 GHz was chosen as this was also the frequency band of operation of existing breadcrumb systems, proposed in [3,4,5,6]. As mentioned in [3], the use of the 2.45 GHz ISM band has as an advantage over lower frequencies that an antenna with an acceptable radiation efficiency can be deployed on a compact breadcrumb node. The Tx SDR was controlled by a computer that was positioned behind a pillar to limit its influence on the environment. The Rx SDR was connected to a laptop that was deployed in the least intrusive way to minimize its impact on the radio wave propagation. This hardware setup was selected to replicate the propagation conditions of one single communication link between two breadcrumb nodes deployed by the RIT during its operation. The Tx and Rx antenna height equaled 10 cm, comparable with the breadcrumbs deployed on the floor.

To characterize the received power in a simple and accurate manner, an unmodulated 2.45 GHz waveform was transmitted. The Rx received 250,000 raw data samples at a time with a receiver noise bandwidth of 25 MHz. An averaging factor of five was applied and subsequently the system calculated the power over the 50,000 averaged samples, after which the Rx was moved to the next data capture point.

### 2.2. Location

**Figure 1:**
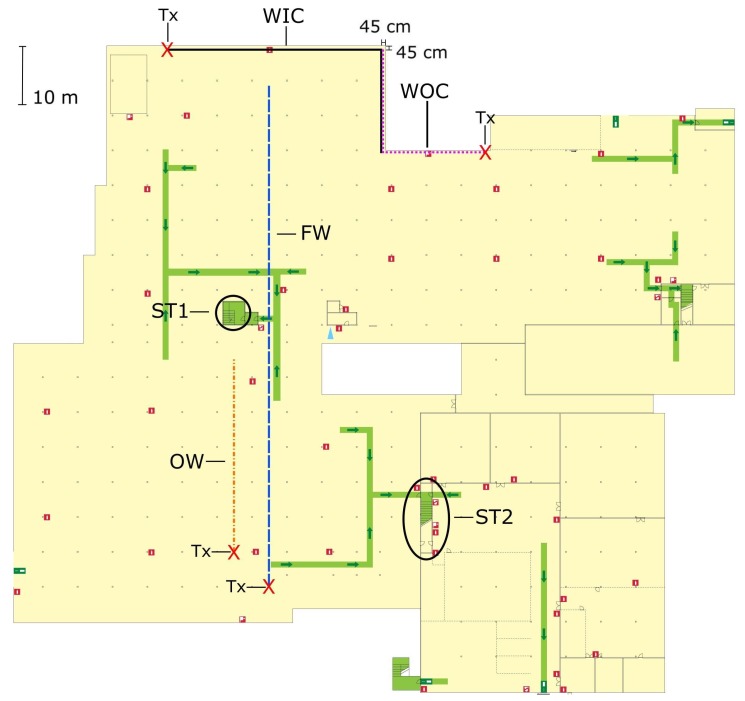
Ground plan of the underground parking garage, showing the different measurement scenarios: dashed blue line: free way (FW), path to and from the parking spots (Figure 2); dash-dotted orange line: obstructed way (OW), path through a line of parked cars (Figure 3); solid black line: along the wall with inside corner (WIC) (Figure 4); dotted purple line: along the wall with outside corner (WOC) (Figure 5); Staircase 1 (ST1, two 90${}^{\circ }$ turns) (Figure 6); and Staircase 2 (ST2, straight) (Figure 7). Transmitter (Tx) locations are marked by red crosses. The green lines with arrows represent evacuation paths for pedestrians in the case of an emergency. It should be noted that these paths are different from the paths that first responders would use, especially in the case of a fire when a path along the walls would be followed.

All measurements took place in the underground parking garage of the P-building on campus Schoonmeersen of Ghent University in Belgium. A ground plan of the area is shown in Figure 1. The scenarios are denoted as Free Way (FW), Obstructed Way (OW), Wall and Inside Corner (WIC) and Wall and Outside Corner (WOC). The radio wave propagation channel between nodes deployed on two different staircases is also characterized, specifically for a staircase with two 90${}^{\circ }$ turns (ST1) and a straight staircase (ST2). During the measurements, the Tx was kept stationary while the Rx was moved along the measurement path.

**Figure 2:**
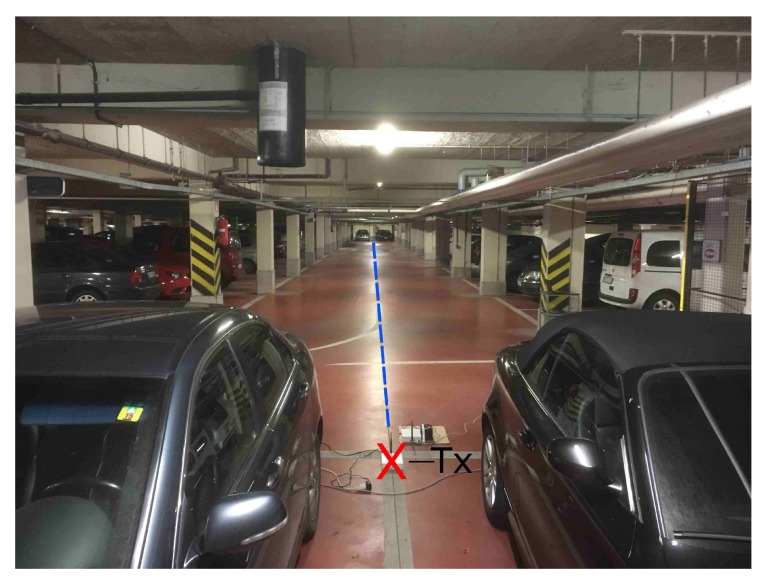
Scenario FW: the Tx location (red X) and the measurement path (dashed blue line ). In this picture, the parking garage is nearly full. Note the different features (such as pillars, water pipes, and ceiling relief) influencing propagation.

**Figure 3:**
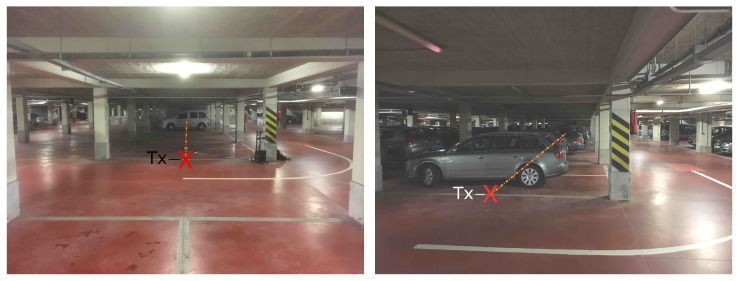
Scenario OW: View from behind the Tx (red X), for the nearly empty underground parking garage (**left**); and the view to the side of the Tx (red X), at the moment when the garage was nearly full (**right**). The measurement path is indicated by the dashed orange line.

**Figure 4:**
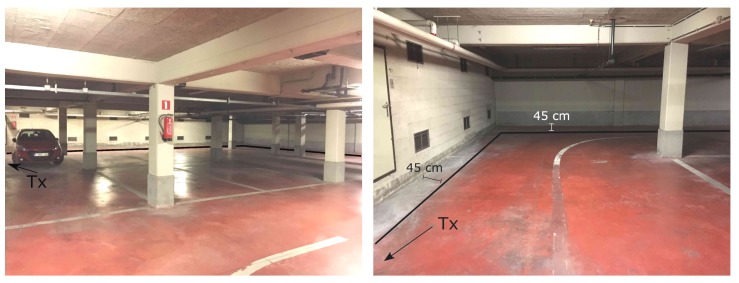
Scenario WIC: View showing the wall and the corner where the measurement takes place (**left**) and a close-up (**right**). The measurement path is indicated by the black line, the location of the Tx is pointed to by the arrow. The underground parking garage was almost completely empty at this time.

**Figure 5:**
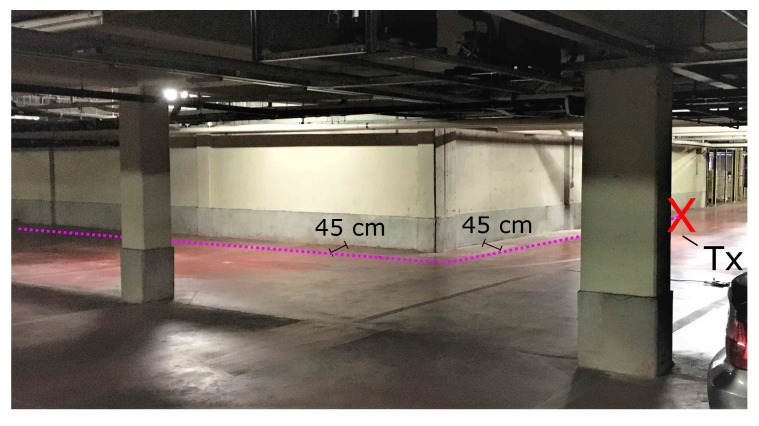
Scenario WOC: View of the outside corner. The Tx is indicated by the red X. The measurement path is represented by the dashed purple line. The underground parking garage was almost completely empty at the time of the measurement.

**Figure 6:**
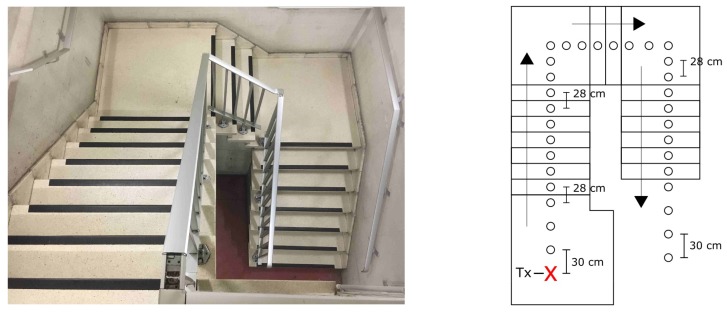
Scenario ST1: Photo of Staircase 1 (**left**); and graphical representation of the measurement points (**right**).

**Figure 7:**
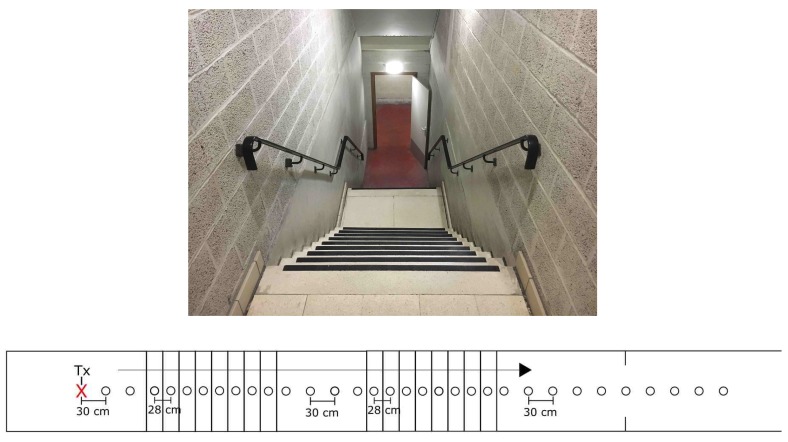
Scenario ST2: Photo of Staircase 2 (**top**); and graphical representation of the measurement points (**bottom**).

The ceiling of the underground parking garage is covered by insulation material in the form of plates consisting of fiber-reinforced plaster. There are water pipes attached to the ceiling and because of this the height of the garage is hard to determine exactly but it can be approximated as 3 m. The walls and floor are mainly comprised of (reinforced) concrete. The structure is supported by an array of square pillars with a width of 40 cm, the positions of these pillars are marked in Figure 1 by gray dots. Visual details are provided in Figure 2, Figure 3, Figure 4, Figure 5, Figure 6 and Figure 7. Because the nature of the underground parking garage dictates that an arbitrary number of cars could be present, some scenarios were measured twice, once for a nearly empty situation (almost no cars present) and another time when the parking lot was nearly full (many cars present), to determine the influence of the parked cars.

### 2.3. Scenario FW

The first scenario offers a continuous LOS path on the road to and from the parking spots. This scenario is marked as FW in Figure 1. No obstacles are present, but some water pipes are attached to the ceiling, which may influence the propagation of the radio waves. Figure 2 provides a more detailed view. A first measurement was executed when the underground parking garage was nearly empty with a rather low spatial resolution (The low resolution is defined as follows: a resolution of 0.3 m for Tx–Rx distances up to 10 m, a resolution of 0.5 m up to 20 m and a resolution of 1.0 m for distances further than 20 m), to increase measurement efficiency. This was repeated for a nearly full underground parking garage. At the low resolution, however, the spacing between points at large Tx–Rx range became too large, and thus averaging to remove the small-scale fading became impossible (see Section 3.1). To mitigate this problem, a third measurement was performed with a higher spatial resolution (The higher resolution measurements have a resolution of 0.05 m throughout), again when the garage was nearly empty.

### 2.4. Scenario OW

Marked as OW in Figure 1, this scenario is similar to the FW scenario but the measurement path runs through the parking spots and is shorter in distance. The last Rx position approaches the opposite wall and here the ceiling is free of water pipes in this location. These measurements were only conducted at a low spatial resolution, for a nearly empty and a nearly full underground parking garage. The first case offers a continuous LOS path, whereas in the second configuration the LOS is obstructed by parked cars. The difference between the two situations is shown in Figure 3.

### 2.5. Scenario WIC and WOC

Indoor fires will often make the visibility go down dramatically. To keep from becoming disoriented in these conditions of near zero visibility, RIT members will follow the walls closely by touch. The use of this strategy was simulated in the WIC and WOC scenarios. In these scenarios, the Tx and Rx always remain at a 45 cm distance from the wall, in accordance to the typical movement of the RIT. Both scenarios were measured at a high spatial resolution. In the WIC scenario, an inside corner is encountered at 37 m. In the WOC scenario, an outside corner is met at 17 m, which means that the LOS component is lost when passing the corner. The measurement paths are marked with WIC and WOC in Figure 1. When processing the data, the distance is taken along this line and described as traveled distance. This means that after passing the corner, the distance is not the shortest path between Tx and Rx but, instead, the total distance traveled. This was done to make the results more applicable to the characterization of a breadcrumb system. These scenarios were only measured when the parking garage was nearly empty. The reasons for this were to isolate the effects of the corner in the measurement path from other influences, such as the presence of parked cars, and because of time considerations, since each high resolution measurement took a lot of time and effort. Nevertheless, doing these measurements in a full parking garage could be interesting as future research. Figure 4 displays the details of the WIC scenario and Figure 5 shows those of the WOC scenario.

### 2.6. Scenario ST1 and ST2

The staircase is a crucial location for an RIT: it serves as an access point, it is used to evacuate people and it is a possible location for an improvised base. The underground parking garage in this research offers two staircases: one with two $90^{\circ }$ bends (ST1) and the other completely straight (ST2). Pictures of these staircases together with a graphical representation of the measurement points are given in Figure 6 and Figure 7.

## 3. Analysis Procedure

### 3.1. Signal Loss

The signal loss at a certain point $x_{i}$ is defined as the ratio of the power of the received signal ($P_{i,\mathrm{received}}$) and the power of the transmitted signal ($P_{\mathrm{transmitted}}$), both expressed in Watt:

(1) $$P_{i}=\frac{P_{i,\mathrm{received}}\left[W\right]}{P_{\mathrm{transmitted}}\left[W\right]}$$

This ratio can be expressed in terms of dB by using the transformation $P_{i}\left[\mathrm{dB}\right]=10\log_{10}P_{i}$.

To make the data easier to interpret and to remove the effects of small-scale fading, the measured data were averaged over a distance of 10λ, with λ being the wavelength of the carrier wave (≈12.2 cm). The resulting averaged data would only be subject to large-scale fading and path loss [12]. The averaging was performed in the following way:

Firstly, for every point $x_{k}$, a set $S_{k}$ was constructed containing all values $P_{j}\left[\mathrm{dB}\right]$ for which the positions $x_{j}$ are within a distance $\frac{10\lambda }{2}$ of the point $x_{k}$:

(2) $$S_{k}=\left\{P_{j}\left[\mathrm{dB}\right]:x_{j}\in \left[x_{k}-\frac{10\lambda }{2},x_{k}+\frac{10\lambda }{2}\right]\right\}$$

Secondly, an averaged value of the signal loss was calculated at the point $x_{k}$ according to the following formula (This formula is analogous to taking the arithmetic mean of the non-logarithmic signal loss *P*):

(3) $$P_{k,\;\mathrm{averaged}}\left[\mathrm{dB}\right]=10\cdot \log_{10}\left(\frac{1}{n(S_{k})}\times \sum_{P_{j}\left[\mathrm{dB}\right]\in S_{k}}10^{\frac{P_{j}\left[\mathrm{dB}\right]}{10}}\right)$$

where $n(S_{k})$ symbolizes the number of elements (It is important to note that $n(S_{k})$ is not the same for every *k* and its value will change when the measurement resolution changes) in $S_{k}$. These two steps were done for every measured point so that the original dataset was transformed into an averaged dataset. Because large-scale fading and path loss were the most relevant channel characteristics for this research, the subsequent analysis was performed on the averaged data only. The reader can find the data in the supplementary files of this paper.

### 3.2. Data Analysis

The data analysis entailed multiple steps. The first step consisted of performing a least-squares fit to extract the path loss index (This is sometimes also called the path loss exponent or loss exponent) (PLI) where applicable [12]. The fitting of the path loss lines was performed over the data points that follow a linear trend (when distance was expressed on a logarithmic scale). To provide an indication of the degree of accuracy on the fitted PLI, a 95% confidence interval of the PLI was constructed. This confidence interval was calculated by using the variance on the PLI estimation and the assumption that the PLI estimation follows a normal distribution. To characterize the large-scale fading, the standard deviation of the fluctuations of the signal loss around the fitted path loss line (${\hat{\sigma }}_{\mathrm{shadowing}}$) was calculated.

The central limit theorem predicts that shadowing causes fluctuations of the received power, expressed in dB, that follow a normal distribution [12]. For every PLI fit, a Q-Q plot was made to evaluate to what extent the fluctuations of the signal loss around the fitted path loss line follow a normal distribution.

## 4. Results

All calculated path loss indices together with their 95% confidence intervals are summarized in Table 1. In the last column, the standard deviation associated with the large-scale fading (${\hat{\sigma }}_{\mathrm{shadowing}}$) is shown. The values obtained by low resolution measurements are placed between brackets because the removal of the small-scale fading from the low resolution data is sub-optimal and, therefore, ${\hat{\sigma }}_{\mathrm{shadowing}}$ is not representative for only the large scale-fading. To make Table 1 complete, the ST1 scenario is also included. However, in this scenario, no path loss line was fitted and thus no values can be given.

### 4.1. Scenario FW and OW

**Figure 8:**
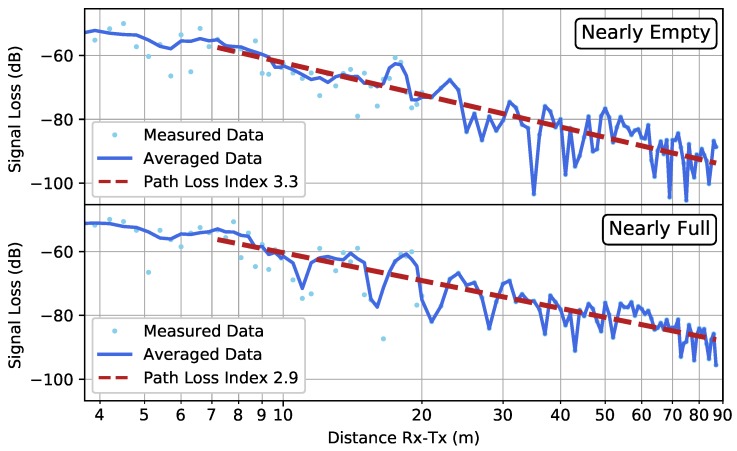
Scenario FW: The low-resolution measured data in the case of a nearly empty parking garage (**top**) and a nearly full parking garage (**bottom**). The average computed by Equation (3) is shown by the solid line, whereas the fitted path loss line is indicated by the dashed line.

Figure 8 shows the results for the two low-resolution measurements within scenario FW. Comparing both datasets, one notices that the measurements performed in the nearly full garage gave rise to a smaller path loss index than the ones in the nearly empty garage. This could be attributed to canyoning by rows of cars that form a waveguide for the electromagnetic wave propagation [13]. Effects of shadowing can be seen at scales of around 10λ (≈1.22 m).

**Figure 9:**
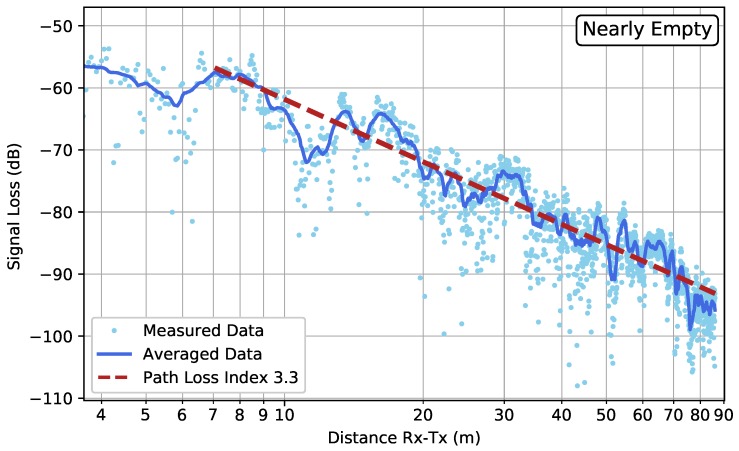
Scenario FW (HR): The high-resolution measured data in the case of a nearly empty parking garage. The average computed by Equation (3) is shown by the solid line, whereas the fitted path loss line is indicated by the dashed line.

Figure 9 provides the results of the higher resolution measurement, performed when the garage was nearly empty. The PLI found in the low-resolution and high-resolution measurements were very similar, demonstrating the reproducibility of the measurements. However, Table 1 shows that the 95% confidence interval was much smaller in the high-resolution measurement, as is to be expected.

**Figure 10:**
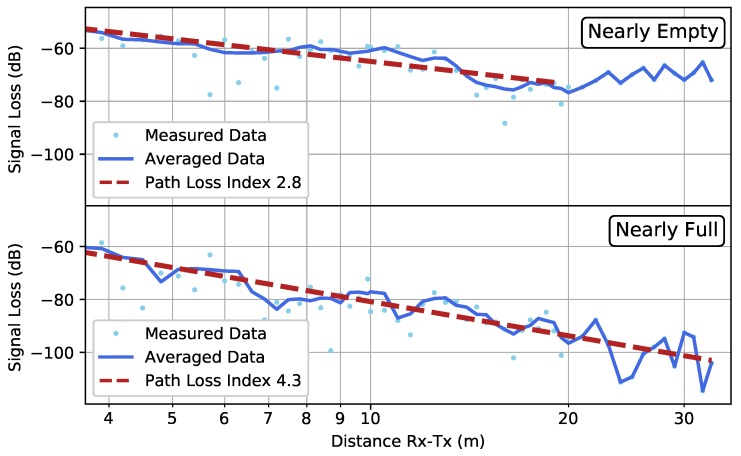
Scenario OW: The low-resolution measured data in the case of a nearly empty parking garage (**top**) and a nearly full parking garage (**bottom**). The average computed by Equation (3) is shown by the solid line, whereas the fitted path loss line is indicated by the dashed line.

The results of the OW scenario are shown in Figure 10. In this case, the difference in PLIs for the nearly full and nearly empty parking garage was much larger than in the FW scenario. Due to the transmitter and receiver being so close to the ground, the cars blocked the LOS component of the connection, which had a detrimental effect upon the PLI. As expected, the PLI that was found in the case of a nearly empty parking garage was comparable in the OW and FW scenarios. The data at larger distance (from 20m onward) in the nearly empty OW scenario were not taken into account for the PLI fit because of their non-monotonous behavior. This behavior could be caused by the reflecting wall at the end of the measurement line, as shown in Figure 1.

### 4.2. Scenario WIC and WOC

**Figure 11:**
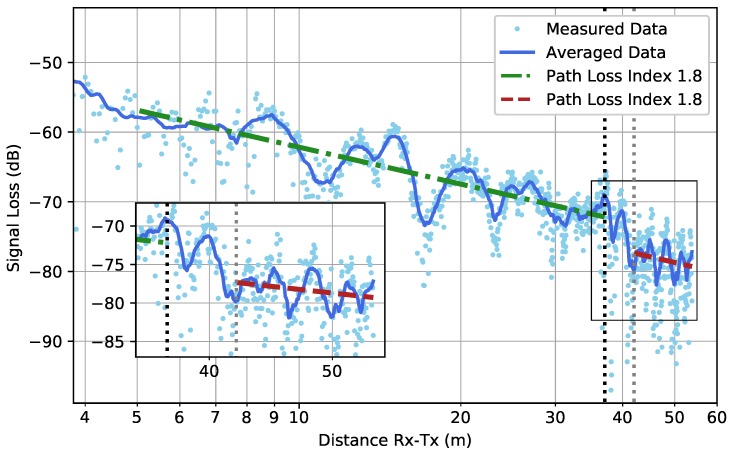
Scenario WIC: The high-resolution measured data together with their averaged values (*solid line*) according to Equation (3). The black dotted line illustrates the position of the corner within the path. The dotted grey line is the point at which the far-zone behavior resumed. Firstly, the path loss was fitted (*dash-dotted line*) on the data until the corner was reached and, secondly, the path loss was fitted (*dashed line*) when the far-zone behavior resumed.

**Figure 12:**
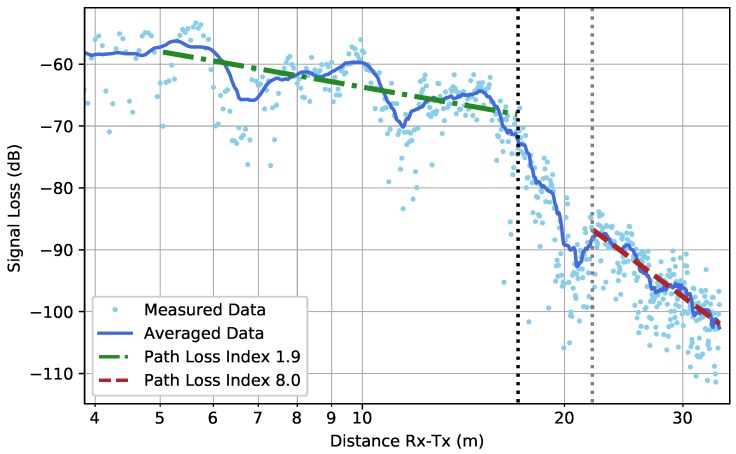
Scenario WOC: The high-resolution measured data together with their averaged values (*solid line*) according to Equation (3). The black dotted line illustrates the position of the corner within the path. The dotted grey line is the point at which the far-zone behavior resumed. Firstly, the path loss was fitted (*dash-dotted line*) on the data until the corner was reached and, secondly, the path loss was fitted (*dashed line*) when the far-zone behavior resumed.

The results from the WIC and WOC scenarios are shown in Figure 11 and Figure 12, respectively. Before the corner was reached, a path loss index of below 2 was found, which indicates a propagation more efficient than in free-space. In both scenarios, the received signal strength dropped very suddenly when the corner was reached, until a certain point at which the far-zone behavior resumed (The actual point at which far-zone behavior resumed is hard to pinpoint and there is a certain degree of subjectivity when determining it. In this research, far-zone behavior was said to resume when a linear trend was again visible). This corner loss effect in the case of an outer corner has already been described for environments similar to underground parking garages in [14]. The corner loss was most pronounced in the WOC scenario due to the loss of the LOS component when turning around the corner. In the WIC scenario, the effect was smaller. To the knowledge of the authors, this effect has not been described in the case of an inner corner.

After this sudden drop when the corner was turned, the far-zone behavior resumed. In the WOC scenario, the PLI increased greatly comparison to the PLI before the corner. In the WIC scenario, however, the PLI remained rather unchanged.

### 4.3. Scenario ST1 and ST2

**Figure 13:**
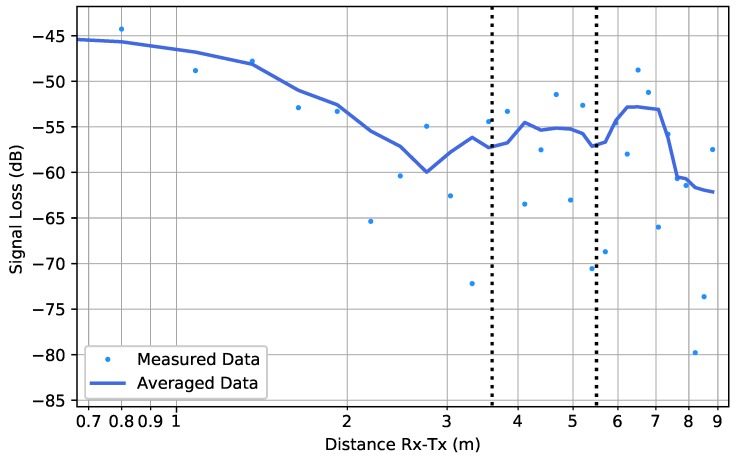
Scenario ST1: The measured data together with their averaged values (*solid line*) according to Equation (3). The two black dotted lines illustrate the position of the two 90${}^{\circ }$ turns in the staircase.

**Figure 14:**
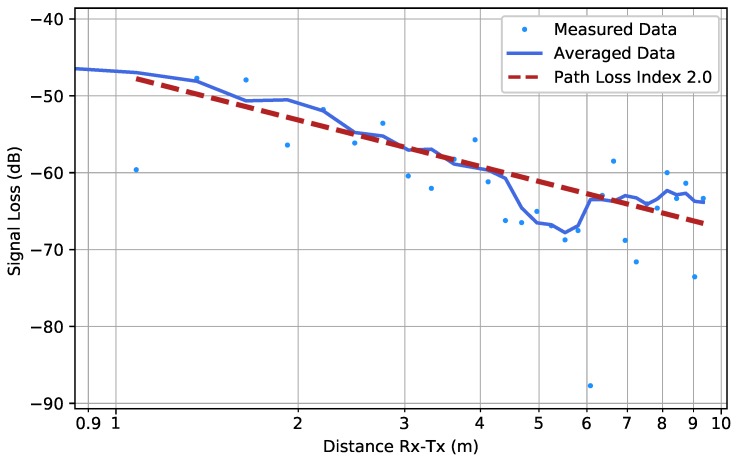
Scenario ST2: The measured data together with their averaged values (*solid line*) according to Equation (3). The fitted path loss line is also shown (*dashed line*).

Figured Figure 13 and Figure 14 show the results for scenarios ST1 and ST2. The two bends in ST1 and the non-linear trend of the data made the fitting of a path loss line meaningless. In the ST2 data, a linear trend was much more clearly visible and therefore a path loss line was fitted for this scenario. The calculated PLI closely approached the free-space PLI of 2. In both scenarios, the total signal loss over the two staircases was around 60dB.

### 4.4. Q-Q Plots

**Figure 15:**
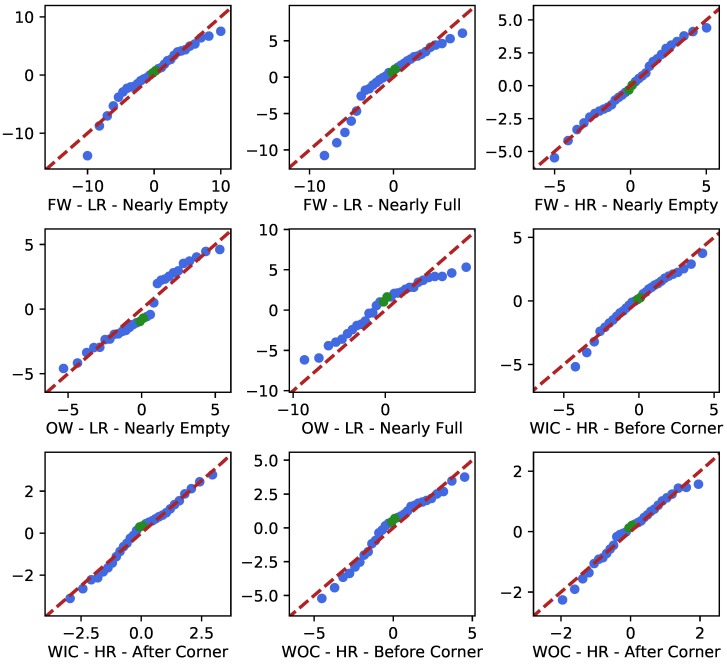
The Q-Q plots of the different scenarios. The blue points correspond to 30 equally large quantiles of the empirical distribution (y-coordinate, in dB) plotted against the same quantiles of the theoretical normal distribution (x-coordinate, in dB). The middle two quantiles are given in green, and the dashed red lines illustrates the first bisector (x = y).

To verify how well the shadowing phenomenon fit a log-normal distribution, in Figure 15, the Q-Q plots of the shadowing fluctuations in the different scenarios are given. The theoretical normal distributions have zero mean and a standard deviation equal to ${\hat{\sigma }}_{\mathrm{shadowing}}$. These results are further analyzed in Section 5.2.

## 5. Discussion

### 5.1. Path Loss

Of all the measurements, the lowest PLIs were found in the WIC and WOC scenarios (before the corners). This implies that following a path along the wall would be the best strategy when using a breadcrumb system. This is in full accordance with current RIT procedures.

Even though the parked cars were not as high as the ceiling of the parking garage, their presence had multiple effects upon the path loss, mostly due to the low position of the breadcrumbs on the ground. From the OW measurements, it seems that the presence of parked cars would have negative effects on the received signal strength when the cars are positioned between the breadcrumb and the corresponding firefighter. From the FW measurements, however, it appears that the presence of parked cars might have positive effects as well, causing a canyoning effect when the radio wave signal propagates between two rows of cars.

In [15], a multitude of path loss indices are reported as they are found in different types of propagation environments and scenarios. Specifically, a path loss index of 1.6–1.8 is reported for indoor environments when an LOS is present between Tx and Rx. The LOS scenarios in this work, being FW, OW—Nearly Empty, WIC and WOC—Before Corner, yielded comparable results, except for FW and OW—Nearly Empty. The larger PLIs that were measured in those scenarios might be explained by the propagation mechanisms specific to an underground parking garage, which differ from the environments considered in [15]. The unusually low position of the Tx and Rx antennas in this research could also play a role in the discrepancy. In the case of non-LOS scenarios, Rappaport et al. [15] reported a PLI between 4 and 6 in building environments. The PLI that was found in the OW—Nearly Full scenario agrees with these reported values.

### 5.2. Shadowing

In most measurements, the effects of shadowing are strongly present. This can be attributed to the various (reinforced) concrete structures, which have large penetration loss and low reflection loss, affecting the radio wave propagation in different manners as the distance between the user and the breadcrumb nodes changes. Shadowing fluctuations can have amplitudes of around 10 dB and a width in the order of 1 m. This effect must be taken into account when implementing a reliable strategy for optimal breadcrumb node deployment, as the (unnecessary) deployment of nodes in a valley of large signal loss would best be avoided. In addition, diversity schemes will have to be implemented to mitigate multipath fading, which was removed from the measurement data by the averaging procedure according to Equation (3).

When analyzing the Q-Q plots, some deviations from normality can be seen. These are mainly present in the low-resolution cases, due to the absence of thorough averaging given the low measurement resolution. This causes a part of the small-scale fading to still be present in the processed data. In the high-resolution results, these deviations are very minor and one can conclude that the large-scale fading is indeed normally distributed (when expressed in dB). The small deviations from the first bisector at the extremities can be explained by the small amount of data points at the distribution tails with respect to the middle.

When looking at the WIC and WOC results in Table 1, ${\hat{\sigma }}_{\mathrm{shadowing}}$ can be seen to be larger before the corner than behind the corner, meaning that the magnitude of the shadowing effect was less significant after the corner was turned.

### 5.3. Corner Loss

Although corner loss is a shadowing phenomenon, it is discussed separately as it is a predictable effect, in contrast to other, random shadowing effects. The sudden loss in signal strength when turning a corner can trigger the deployment system to drop a new node. Needless to say, to deploy a node after the corner would be sub-optimal and it would be better to anticipate the corner loss by deploying a node at the position of the corner. It would, however, not be easy for the system to detect the turning of a corner and thus a manual override of the deployment mechanism would have to be considered.

### 5.4. Signal Loss over Staircases

The total path loss over the two staircases was around 60 dB. Considering this loss, it would be sufficient to place a breadcrumb node at the beginning and at the end of the staircase while ensuring a reliable communication link [3,7]. However, to improve reliability of the wireless communication channel, higher link redundancy may be foreseen by deploying additional nodes on the staircase.

## 6. Conclusions

In an underground parking garage, the smallest amount of path loss will be suffered when following a path close along the walls. Along this path, however, one has to be mindful of significant signal loss when turning corners, especially outer corners. When using an automatic breadcrumb system, a dedicated deployment algorithm must be put in place to anticipate these corner losses, or a manual override must be implemented and RIT members must be made aware of the hazards of losing communication due to corner losses. Furthermore, due to the properties of the building materials used in underground parking garages, in addition to important multipath fading, important shadowing effects occur, which must be carefully taken into account during the implementation of a breadcrumb system. In particular, shadowing requires careful positioning of the different breadcrumb nodes and the deployment of nodes in valleys of large signal loss should be avoided. Moreover, the use of redundant nodes to ensure sufficient reliability of the wireless channel can be beneficial in this type of unpredictable environment. In addition, the measurement results demonstrate that operations over multiple floors are possible by bridging the stairwells by only one breadcrumb-to-breadcrumb link (assuming a link redundancy of zero). However, again, it is advised to deploy some additional breadcrumb nodes for redundancy and added reliability. Lastly, by analyzing the statistics, it was found that the variations in signal strength due to large-scale fading, when expressed in dB, are normally distributed.

## Figures and Tables

**Table 1:** The fitted path loss indices together with their 95% confidence intervals and the standard deviation due to the shadowing.

Scenario	Path Loss Index	95% Confidence Interval	${\hat{\sigma }}_{\mathrm{shadowing}}$ (dB)
FW—Nearly Empty—Low Resolution	3.3	[2.9, 3.9]	(5.4)
FW—Nearly Full—Low Resolution	2.9	[2.5, 3.3]	(4.5)
FW—Nearly Empty—High Resolution	3.3	[3.3, 3.4]	2.7
OW—Nearly Empty—Low Resolution	2.8	[2.4, 3.2]	(2.9)
OW—Nearly Full—Low Resolution	4.3	[3.8, 4.8]	(4.7)
WIC—Before Corner—High Resolution	1.8	[1.7, 1.8]	2.3
WIC—After Corner—High Resolution	1.8	[1.1, 2.5]	1.6
WOC—Before Corner—High Resolution	1.9	[1.7, 2.1]	2.4
WOC—After Corner—High Resolution	8.0	[7.7, 8.4]	1.0
ST1—Custom Resolution	-	-	-
ST2—Custom Resolution	2.0	[1.7, 2.3]	(2.6)

## Supplementary Materials

The supplementary materials are available online at https://www.mdpi.com/1424-8220/19/11/2431/s1.

## Abbreviations

The following abbreviations are used in this manuscript:

RIT	Rapid Intervention Team	FW	Free Way
NI	National Instruments	OW	Obstructed Way
LOS	Line of Sight	WIC	Wall and Inner Corner
PLI	Path Loss Index	WOC	Wall and Outer Corner
SDR	Software Defined Radio	LR	Low Resolution
USRP	Universal Software Radio Peripheral	HR	High Resolution
